# The Use of Online Health Forums by Patients With Chronic Cough: Qualitative Study

**DOI:** 10.2196/jmir.7975

**Published:** 2018-01-24

**Authors:** Ashnish Sinha, Tom Porter, Andrew Wilson

**Affiliations:** ^1^ Norwich Medical School University of East Anglia Norwich United Kingdom

**Keywords:** cough, chronic disease, Internet, eHealth, health impact assessment, information systems, help-seeking behavior, social support

## Abstract

**Background:**

Online health discussion forums are used by different patient groups for sharing advice and information. Chronic cough is a common problem, and people with chronic cough use online health forums alongside formal medical therapies.

**Objective:**

The objective of this study was to assess how chronic cough sufferers use online health forums, including the treatment advice they share with one another and the possible clinical uses of online forums in chronic cough.

**Methods:**

Three open-access health forums were searched for threads related to chronic cough. Identified threads were screened against inclusion and exclusion criteria adapted from the British Thoracic Society (BTS) Guidelines related to chronic cough diagnosis. Included data were subjected to qualitative thematic analysis. All study data were cross-validated by a second author and discrepancies were resolved.

**Results:**

In total, 96 threads were included in the analysis, consisting of posts by 223 forum users. Three main themes were identified: the effect of chronic cough on the lives of patients, the treatment advice shared between users, and the provision of support within forums.

**Conclusions:**

Chronic cough symptoms had impacts on multiple aspects of patients’ health and well-being. To try and combat these issues, forum users suggested a variety of treatments to one another, ranging from mainstream traditional therapies to odd alternative remedies. The provision of support and empathy were also prominent themes in discussion threads. Online forums themselves may provide increasing benefit to users through the addition of a moderator.

## Introduction

### The Internet in Health

An ever-increasing number of patients are gaining access to the Internet and harnessing the wealth of information it contains. A 2011 report by the World Health Organization (WHO) estimated that 2 billion people across the world had Internet access, and the current number is likely to be vastly higher [1]. The Internet is a widely available source of health-related information, and [2,3] recent research has also suggested that around 70% of those using the Internet have used it to search for health information, highlighting the high demand for medical information by the public [4].

### The Burden of Chronic Cough

People with health complaints routinely use online health forums when seeking information [5]. Chronic cough, defined as a cough which is persistent for more than 8 weeks [6-8], is featured in online health forums. It has an estimated prevalence of 12.7% in Europe and 11.0% in the United States [9]. Yet, the cause of patients’ chronic cough often remains undetermined, limiting the scope for clinicians to provide effective treatments [6-8]. Chronic cough has been shown to have an impact on the quality of life (QOL), which has been described in relation to the biopsychosocial model of health [10,11]. Studies have also shown that chronic cough is associated with poorer health-related quality of life (HRQOL) in the domains of social interaction, sleep, and work [12-14], illustrating the potential for chronic cough to disrupt the lives of those whom it affects. Chronic cough also confers burdens beyond the individual, as in the case of parents of children with chronic cough, who report stress and anxiety [15]. The combination of debilitating symptoms and minimal support availability from clinicians often leads patients to seek alternative advice regarding therapies and support, utilizing online forums as a medium for discussion.

### Online Health Forums

Online health forums are a source of health information, providing patients with a safe environment to share experiences, seek information, and improve their health knowledge [16-18]. Health forum users have been shown to benefit from online interventions, resulting in greater knowledge about their conditions and greater health activation, with similar efficacy to non-Internet interventions [4,16]. Alongside this, forums provide important opportunities for social support, reassurance, and friendship [18,19]. Recent studies have detailed the content of the information shared in online forums, highlighting its accuracy and identifying the importance of language in promoting discussion [20-23]. The moderation of forums, whereby clinicians or forum staff access the forums and remove harmful posts and avoid repetition, has also been explored [18,24]. Overall, online discussion forums have been shown to be beneficial for users, indicating that further research into their nature and possible utility in medical care is warranted [4,25].

To date, health forum studies have predominantly focused on mental health conditions, but recent research has investigated chronic conditions such as type 2 diabetes mellitus, breast cancer, and stroke [26,27]. Despite its presence in forum discussions, research is yet to explore the use of online health forums in relation to chronic cough. This qualitative study aimed to explore how people living with chronic cough engage with, and make use of, online health forums.

## Methods

The study was conducted as a qualitative exploration of three large, open-access online health forums. Forums were identified through a simple search term of “cough health forum” in an online search engine, as in previous studies [19,22]. One author identified threads related to chronic cough within the forums, using a further search term of “chronic cough.” Threads were screened against inclusion and exclusion criteria adapted from the British Thoracic Society (BTS) Guidelines on chronic cough management [8]. Included forum threads were required to be posted in English language and satisfy the criteria for chronic cough. Threads were excluded if patients’ posts suggested an alternative diagnosis or the duration of cough was not specified (Multimedia Appendix 1). This process was continued until data saturation, which was recognized when no new themes were emerging from the coding. We focused particularly on threads discussing chronic cough of unknown etiology, as diagnostic uncertainty is recognized to have an impact on the lived experience of a disease in patients [28,29]. Data were collected between October 2015 and January 2016. The threads themselves ranged in dates from 2002 to 2015 across the three forums, with users still actively participating in these discussions at the time of data collection.

Included forums’ threads were transcribed verbatim into the NVivo 11 program (QSR International Pty Ltd. Version 11, 2015). Threads were analyzed using thematic analysis, with a single author first coding the data into large themes [30]. The data were subsequently coded into subthemes to improve the depth of analysis. The themes and subthemes identified by the primary author were discussed and defended with the other study authors. Then, a secondary author reviewed the threads for the accuracy of coding, but did not recode the data. Discrepancies identified between the study authors were discussed and consensus was reached.

Ethical approval for the study was granted by the host institution’s research ethics committee. Ethical issues were limited by using open-access forums as per previous explorations of Internet research ethics [31-33]. Though previous research has suggested that it is difficult to maintain anonymity in online health forums, the usernames of users posting on the forums were removed, and a unique identifier was given [34]. The authors had no contact with any forum users; thus, no respondent validation could be performed. Risks to forum users from the research were deemed to be minimal.

## Results

**Figure 1:**
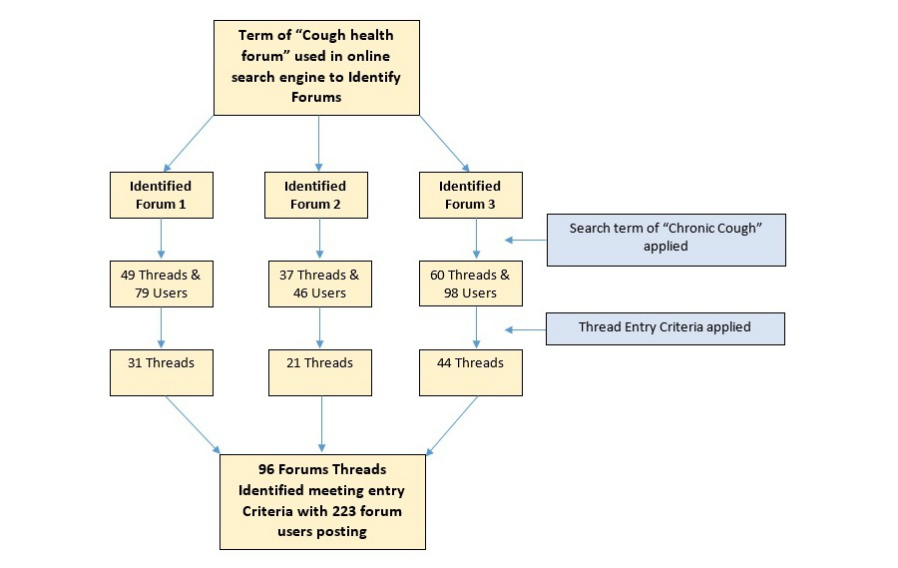
Flow diagram of the process for thread identification.

Figure 1 shows that from the total 223 users, 3 were known medical professionals who were identified because of their usernames and an image from the forum, whereas 1 user in another forum claimed to be a doctor. Moreover, 2 nonmedical moderators were present in forum discussions, and each of them posted in several threads.

### Findings

Forum posts consisted of an initial comment or question posed by an original poster (OP), followed by responses and discussion involving responding posters (RP). OP posts not only illustrate the physical impact of chronic cough but also highlight psychological and social consequences. In response to OP posts, RPs offered support in various forms and demonstrated lay knowledge derived from the lived experiences of chronic cough, as well as their appropriation of biomedical knowledge. Forums were a source of distinct forms of support, including suggestions as to traditional and prescribed drug treatments, home remedies, lay referrals, as well as social and emotional support. The following sections outline each of these in turn.

### The Impact of Chronic Cough

#### Physical Consequences

Cough is often seen as an innocuous symptom, yet multiple OPs described adverse physical sequelae resulting from coughing. Of the 96 threads analyzed, 26 contained accounts of physical impairments directly relating to cough.

The most commonly reported symptoms included bodily aching and vomiting, which were mentioned by 6 and 5 users, respectively. Threads illustrate clearly the distressing and disruptive nature of physical complaints, as User #136 posted: “I am at my wits end,” adding “my ribs and my back are very sore from all the coughing.”

Another forum user described their spouse’s struggle with physical symptoms:

...the minute he gets up he coughs until he vomits many times.

User #133

Several users detailed yet more debilitating symptoms, including urinary incontinence (2 users) and hoarseness (2 users). Forum users’ descriptions of these complaints illustrate just how disruptive the physical consequences of chronic cough may be; one OP said:

Last year the cough was so severe I fractured two ribs.

User #001

Another user posted that:

[He] coughed so hard that I have developed costacondritis [sic] (cracked rib cartilage) twice and ended up in the emergency room, adding that now, I have incontinence [sic] so bad, that I have to urinate every hour.

User #158

#### Psychological Consequences

Adverse physical complaints resulted in psychological distress for many users, with posts detailing how persistent symptoms led to feelings of low mood and frustration. Sleep disturbance, tiredness, and lethargy were also common. One user posted as follows:

Now it is a constant cough, is intensified at night (all the night), I can't sleep. Therefore I am very tired, with anxiety, irritable, desperate.

User #097

Another user posted succinctly:

I feel down, lethargic fatigue

User #199

In more extreme instances, forum users said they felt “desperate,” and several posters expressed anger toward confidants, with numerous posts detailing frustrating interactions with both family members and health care professionals. One user posted the following:

I keep telling my parents to take me to the doctor because I've had this thing for two years, but they always roll their eyes and tell me that I'm making a big deal out of nothing and they think I'm lying. They don't have to live with feeling sick 24/7. They don't know...Someone please tell me what I should do. I really can't take this any longer.

User #046

#### Social Consequences

Several users highlighted the impact of coughing on social relationships and daily activities, with symptoms precluding certain social activities and settings. One user posted the following:

I stopped going to church because people look at me like I have something really bad. Sometimes I throw up a lot of mucous and I really cough a lot at night...Sometimes I wet on myself I cough so hard and it makes my head hurt. I can't go anywhere without fear of having these attacks...I've become withdrawn from society.

User #128

The following post demonstrates clearly the loss of control and uncertainty delivered by chronic cough: *I can't go anywhere without fear of having these attacks.* In this example, the forum user is concerned about how their symptoms may be perceived by others, which leads them to withdraw from a previously valued social activity. Such social activities and social networks are important domains of QOL, and it is easy to envisage how this forum user’s well-being would suffer because of this social withdrawal.

Some OPs were family members posting on behalf of a relative (included parents of children with cough). User #106 was one such parent, whose post detailed experiences of caring for a daughter with cough:

My little girl has had an asthma sounding cough for a year on and off. I'm at my wits end as it goes for a week or so comes back for two weeks, In fact I'm pretty sure we have not had a month without coughing, in particular at night. She wakes up coughing at least 3 times at night.

This forum user continued to explain the impact of their daughter’s symptoms, which further emphasize the social consequences of chronic cough:

I'm exhausted with it and so is she bless her...I'm on the verge of quitting my job as I can't cope with being up at night and then going to work and I love my job...I feel very down with this and my partner and I argue all the time as we are both tired and things in the house are getting on top of us.

User #106

As this excerpt suggests, chronic cough disrupts normal patterns of everyday life, which in turn affect the functioning of social roles and relationships. This forum user explains that her daughter’s cough has resulted in strained relations with her partner while also making her own prospects of employment less viable.

### Lay Knowledge

In response to original posts, RPs provided various forms of support, including traditional and prescribed drug treatment suggestions, home remedies, and lay referrals. Ensuing threads demonstrate users drawing upon lay knowledge—garnered through the lived experiences of chronic cough and care—to inform, advise, and support.

#### Biomedical and Prescribed Drug Treatments

In total, forum users recommended 40 differed drug treatments. These recommendations encompassed a variety of treatments ranging from inhalers, such as beta-2 agonists, to antibiotics, and also highly particular treatments for specific conditions, such as Pirfenidone (a treatment for idiopathic pulmonary fibrosis). One user posted the following:

It could be rhinitis—allergies. Try taking an antihistamine tablet each night, such as citirizine or loratidine. This worked for me.

User #036

This reply is typical in that the forum user draws on their own experience of cough and a successful therapy to make a recommendation to an OP. Moreover, this recommendation is premised upon an inferred diagnosis—rhinitis—which the forum user believes to be an underlying cause of the OP’s symptoms. Such assumptions regarding underlying diagnoses were common and often formed the basis for recommended drug treatments. Another example was provided by a user who posted:

You may want to see if you respond to an inhaler, and if you do, you definitely have asthma. Asthma has various triggers, so if you can find your triggers, you'll know how to handle the situation.

User #104

In other threads, the most common diagnoses offered by forum users were common respiratory conditions, namely, asthma, pneumonia, and pulmonary fibrosis. Appraising the quality of forum users’ advice is difficult, but it appears that forum users hold a high level of health knowledge. In most instances, users made sensible suggestions about underlying diagnoses for chronic cough. Furthermore, the advice offered for the amelioration of symptoms was of a good quality, with 33 of 40 recommendations mentioning treatments that were included within the medical guidelines for conditions which commonly cause chronic cough.

#### Home Remedies

Biomedical treatments comprised the majority of recommendations, yet forum users also suggested home or alternative remedies. The content of home remedies varied widely, but often included specific therapies, such as omega therapy, branded alternative remedies, such as Virastop, and nonprescription tablets or sweets. One user provided a typical example of such recommendations:

...so a friend of mine said why dont [sic] you take him to see a omega therapist no medication just healing after 3 sessions we found xx(son) wasnt [sic] coughing as much and after a few months the cough had gone completely (not joking) and the cough never came back, we sat with xx(son) the whole time of his treatment and the lady was just putting her hands on his chest when we asked xx(son) what was happening he said it was like a warm feeling going through his chest, ok we had to pay £20 a session for one hour but it was well worth it.

User #020

Other less conventional recommendations included the application of Vicks vapor rub to the soles of the feet. This particular remedy was recommended by 2 users, one of whom posted:

I’ve [sic] recently found out about rubbing a spot of vapour rub/Vicks on the soles of a child's feet at bedtime to stop them coughing. Well I thought I would share this trick as its a miracle and really works. I used it 3 nights in a row on my 6 year old son and he didn't cough all night.

User #051

Home remedies were often a point of discussion among forum users who drew on their own experience of cough and of particular remedies to validate or question the efficacy of suggested treatments. Home remedies were generally greeted with enthusiasm by OPs, perhaps due to their unconventional nature and the fact that OPs had often exhausted other conventional means of therapy without resolution and with persisting unexplained symptoms.

#### Lay Referrals

Many forum users (46) recommended OPs to consult their doctor or health professional rather than attempting to provide treatment suggestions or diagnoses of their own. Such referrals often included suggestions of potential diagnoses or therapies, which were intended to establish a new line of clinical inquiry. One user posted the following:

Since you have a teaching job and have spent long hours speaking then it would be advisable to consult an ENT specialist to rule out a vocal cord nodule/growth.

User #127

Another user posted the following:

Hi User 026, Catch these things early. See your Doctor, slight chance of thrush. We are NOT Doctors. Good luck & Seasons Greetings.

User #027

As these examples show, lay referrals often affirmed the expertise of professionals while recognizing the limitations of lay knowledge. Unlike standard clinical interactions, such as doctor-patient consultations, online health forums operate without safeguarding frameworks, meaning that forum users can act and communicate in an entirely unrestricted way. Despite this freedom, lay referrals demonstrate an ethic of self-regulation and an awareness that false or misleading health information may cause harm. Thus, users often stressed the contingent nature of their knowledge and advice, and they regularly referred OPs to the appropriate health professionals.

#### Social Support

A significant proportion of RP posts involved aspects of social support. Supportive posts were especially common in response to OP posts that displayed distress or which depicted difficult personal or social circumstances.

Supportive posts often invoked shared experiences as a means of displaying empathy and solidarity with OPs. One user posted the following:

Hi, sadly it took me six months before I was over it, hope your [sic] soon feeling much better.

User #012

Another example was provided by a user who said:

Everyone has given you some great advice here. My stress level would be high too if I were going through what you are going through. My heart goes out to you!”

User #061

Such posts are intended to give OPs hope that their symptoms would abate or that unexplored therapeutic avenues might resolve their troubles. Supportive posts also illustrate a clear affective connection between RPs and OPs, and support previous research that shows online health forums to be important communities of practice.

Beyond shared experiences, a small number of forum users used humor as a tool to alleviate distress expressed by original posters. One user replied to an original post containing a typo:

Secondly if you really are 355 years old it's no wonder you feel bad...hehehe [sic].

User #200

## Discussion

### Study Findings

In this qualitative study of online health forums for chronic cough, we described in detail the multiple adverse physical, psychological, and social effects that were caused due to chronic cough in patients. Subsequently, we explored the treatments and advice suggested by responding forums posters, providing examples of the biomedical and home remedies that users recommended to one another, alongside the idea of lay referral. Finally, we detailed the role of online forums in the provision of support for chronic cough sufferers.

From our analysis, it was evident that chronic cough had a significant impact on the lives of forum users. Its effect was a prominent theme throughout a large number of forum posts. Users highlighted varying levels of physical, psychological, and social impairments, showing that cough symptoms affected all elements of the biopsychosocial model of health; this fits with the previously published research [10,11,14]. Our study supplemented this body of literature by providing an in-depth look at the issues patients were experiencing. Currently, the psychological impacts of cough are more fully documented than the physical consequences. The information we provide details the common physical health consequences of chronic cough, allowing a greater understanding of the symptoms which patients find most concerning. Clinically, this is important as physicians often have few treatments they can provide to combat cough itself. Understanding the subsequent problems provides the opportunity to control these secondary sequelae, improving HRQOL for sufferers. This will also serve to alleviate the frustration that chronic cough patients harbor for medical professionals, who have been unsuccessful in treating their symptoms.

This study also explored the social impacts that cough had on patients and their support network. Users described changing or avoiding their normal activities, as their symptoms induced discrediting stigma. Changes in social activities have previously been reported in relation to chronic cough, whereas stigma has been explored in relation to cystic fibrosis, highlighting cough as a prominent source [11,35]. Our study found similar discussion threads in online health forums with users describing severe limitations to their hobbies or regular activities due to the stigma of their cough symptoms. We also acknowledge the wider impacts of cough, relating to patients’ family and friends. This is recognized in other chronic conditions under the family systems theory [36]. There were multiple instances of family members posting concerns for relatives. Similar findings have previously been described [11,14]. Meanwhile, we also found instances of parents posting for their children, demonstrating the parental stress described by Marchant et al [15]. It is important to recognize that the effects of chronic cough extend beyond the sufferer, often impacting their family and support networks. For clinicians, recognizing chronic cough patients’ concerns and providing appropriate support will help to alleviate the impact on patients as well as the downstream effects on their family and friends.

Help seeking and the provision of advice were prominent in our data. The seeking of health information through online forums is well documented, and the current evidence suggests that users benefit from this information [4,25]. Accordingly, work on health forum posts has attempted to gauge the accuracy of the information that users provide. Previous evidence suggests that the information provided on health forums is of a good quality, even matching the knowledge of clinicians [22,23]. The analysis in our health forums supported these conclusions. Diagnosis suggestions were common conditions which cause cough, such as asthma or reflux disease. Subsequent treatment suggestions matched these diagnoses, indicating that the information was of good quality. Instances of forum users advising others to reconsult their doctor also highlighted an element of insight and responsibility. It ensured that incorrect information was not provided while also encouraging users with severe symptoms to be assessed by a health professional. With the increasing use of health forums, it is important for clinicians to understand both the good and bad information being shared online. This includes the potential for harmful posts to be left either intentionally through a lack of knowledge from RPs or stemming from an insufficient history being provided by OPs. A proposed method of ensuring forum post quality is the use of medical professional–moderated forums. These have previously been trialed and may allow clinicians to provide accurate information to patients, outside of the normal appointment system [18,37]. Chronic cough is a condition with which patients often visit their doctor multiple times. Our data show forum users avoiding doctors’ appointments after bad experiences or lack of effective treatments, citing them as a waste of time. These patients are lost to follow-up in the medical system, but may frequent online health forums, seeking advice from other sources. Directing them toward a moderated forum, where common questions and concerns are answered by a health professional, could prove time-saving for both the patient and doctor, while also ensuring that they remain engaged with health services and are provided correct, up-to-date information.

In our forums, the suggestion of alternative or so-called home remedies was less prominent than the sharing of traditional treatment advice. Yet, it proved an important facet in forum threads, with multiple users corroborating the suggested treatments. These features have previously been recognized in other studies, which describe the behavior of seeking alternative remedies in online forums, as well as their potential harms [38,39]. In our dataset, the most commonly suggested and corroborated treatments were natural remedies such as herbal oils or suggestions of using alternative medicines. Though alternative remedies are often touted to be effective, it is difficult to justify their use due to uncertainty about their mechanism of action, the extent of the placebo effect, and their potential adverse effects. But clinically, it is important to recognize that patients may try alternative therapies, if traditional treatments have had little efficacy. Our study supplements the current literature by identifying some of the alternative therapies that patients are trying for chronic cough. These are important to identify in case of harmful treatments or potential interactions with prescribed medications. There is also the potential to identify alternative therapies which are effective in alleviating cough symptoms.

The final eminent theme identified was the provision of support through online health forums. Numerous users tagged supportive statements to longer posts or posted purely to convey sympathy or empathy for the OP. Previous research has explored the provision of support in health forums, quantifying the number of supportive posts and their nature [32,40,41]. In our forums, the proportion of supportive posts was outweighed by the number of informative posts fitting the previously reported trends. We also identified the methods used to convey this support. Users often recounted their own similar experiences when conveying empathy, a mechanism identified in other health forum studies [19,32]. Humor was very rarely used, highlighting that online health forums for cough are a platform for serious discussion. In the wider literature, this is reflected by a paucity of evidence for humor in online health forums. Chronic cough sufferers are impacted heavily by their symptoms and require support in dealing with them. Understanding the issues they face allows medical professionals to tailor their services toward these issues, providing more effective support. A moderated forum where these ideals may be performed represents an attractive prospect. It would allow for serious discussion about patients’ symptoms while permitting medical professionals to support patients where possible, and it would also help to prevent irrelevant or incorrect posting which previous authors have shown concern about [38].

### Limitations

Our use of open-access, English language, online health forums introduces an element of selection bias due to the prerequisite English language and computer literacy skills required to partake in forum discussions. This also restricts the transferability of our results to subscription-based online health forums. Though the demographics for health forums have been previously reported, we are unable to be sure of how well our forums represent this reported demographic.

Methodologically, we recognize that our search terms for both forum and thread identification alongside our inclusion and exclusion criteria may have resulted in forums or threads related to chronic cough being missed from our analysis. We purposefully excluded chronic related to specific conditions, such as asthma and reflux disease, in our threads, meaning our analysis deals with chronic cough of unknown etiology only. Due to the anonymous nature of posting, we are also unable to corroborate information that is provided, perform triangulation through another data type, or perform respondent validation.

### Conclusions

Chronic cough is a widely discussed topic in health forums. Forum users often detail the large impacts that cough has on their daily lives, encompassing all domains of the biopsychosocial model. Clinically, this information may help health professionals to provide therapies for either the cough itself or its sequelae. Information seeking and provision represents key facets of forum use. Cough sufferers often visit forums seeking advice about a possible treatment or diagnosis, with other users providing treatment suggestions or advising reconsultation with a doctor. Moderated forums, where health professionals provide accurate information, are an alternative, proving superior to repeated consultations.

## Appendix

Multimedia Appendix 1 Table of inclusion and exclusion criteria as well as a summary of the main themes and subthemes explored in the paper.

## Abbreviations

BTS: British Thoracic Society
HRQOL: health-related quality of life
OP: original poster
QOL: quality of life
RP: responding poster
UEA: University of East Anglia
WHO: World Health Organization
