# Supplementary material for: The Use of Online Health Forums by Patients With Chronic Cough: Qualitative Study
Source: J Med Internet Res. 2018 Jan 24;20(1):e19. doi: 10.2196/jmir.7975 (PMC5803533; doi:10.2196/jmir.7975)
Supplement: Multimedia Appendix 1 [file jmir_v20i1e19_app1.pdf]

**Inclusion Criteria**	**Exclusion Criteria**
**Thread contained within a selected Online health forum**	**Symptoms such as hemoptysis which suggest a more serious diagnosis than chronic cough**
**Original thread post specifies a cough of greater than 8 weeks' duration in accordance with the British Thoracic Society guidance on Chronic Cough**	**No specification of the length of cough symptom or a duration of less than 8 weeks**
**Forum text is posted in English Language**	**Forum Text posted in a Non-English Language**

Table 1. Summary of inclusion & Exclusion criteria for online forum threads

Main Theme	Associated Subthemes
**The Impact of Chronic Cough**	Physical impacts
	Psychological impacts
	Social impacts
**Lay Knowledge**	Biomedical and Prescribed drug treatments
	Home remedies
	Lay referral
**Social Support**	

Table 2. Summary of Themes and sub-themes discussed.
